# Supplementary figures and images for: Characterization of Key Odorants in Lushan Yunwu Tea in Response to Intercropping with Flowering Cherry
Source: Foods. 2024 Apr 19;13(8):1252. doi: 10.3390/foods13081252 (PMC11049266; doi:10.3390/foods13081252)

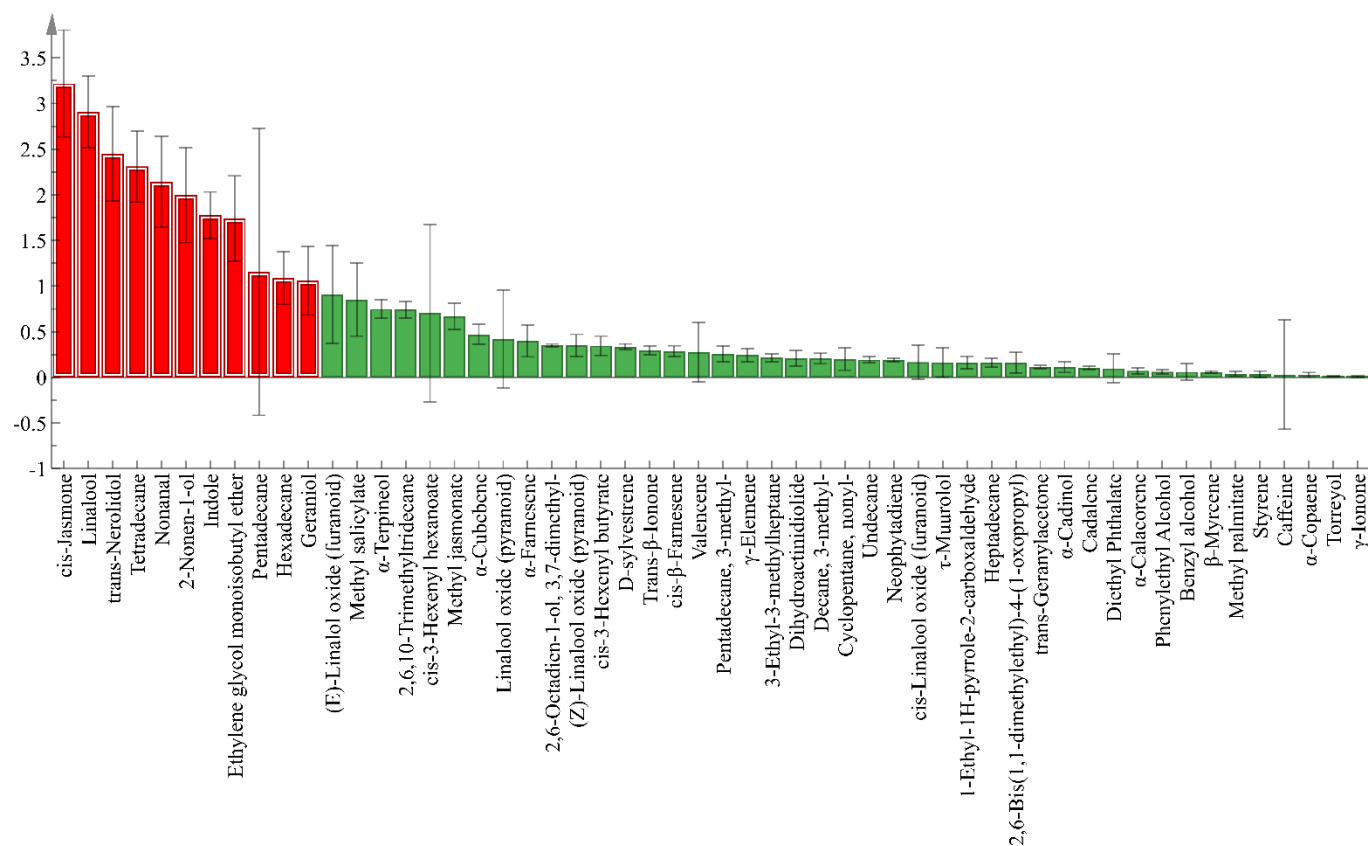

**Figure S1.** VIP values of the 54 volatile compounds of EG and CG green tea samples.

Supplement: Supplementary file 1 [file foods-13-01252-s001.zip › foods-2958910-supplementary.pdf]
